# Overcoming barriers in cognitive assessment of Alzheimer's disease

**DOI:** 10.1590/S1980-57642014DN82000002

**Published:** 2014

**Authors:** Mario Alfredo Parra

**Affiliations:** 1 MD, PhD. Centre for Cognitive Ageing and Cognitive Epidemiology, Human Cognitive Neuroscience, Department of Psychology, University of Edinburgh, UK; Scottish Dementia Clinical Research Network, NHS Scotland, UK; Alzheimer Scotland Dementia Research Centre, University of Edinburgh, UK; Neuroscience Group, University of Antioquia, Colombia; UDP-INECO Foundation Core on Neuroscience (UIFCoN), Diego Portales University, Santiago, Chile.

**Keywords:** Alzheimer's disease, neuropsychological evaluation, cognitive assessment, diagnosis

## Abstract

Diagnosis of Alzheimer's disease (AD) requires a reliable neuropsychological assessment, but major barriers are still encountered when such tests are used across cultures and during the lifespan. This is particularly problematic in developing countries where most of the available assessment tools have been adapted from developed countries. This represents a major limitation as these tests, although properly translated, may not embody the wealth of challenges that a particular culture poses on cognition. This paper centers on two shortcomings of available cognitive tests for AD, namely, their sensitivity to the educational background and to the age of the individual assessed.

## Abstract (pt)

O diagnóstico da doença de Alzheimer (DA) necessita de uma avaliação neuropsicológica de confiança, barreiras importantes ainda são encontradas quando esses testes são usados em através de culturas e durante a vida. Isto é particularmente problemático em países em desenvolvimento, onde a maioria das ferramentas de avaliação disponíveis foram adaptadas a partir de países desenvolvidos. Isto representa uma grande limitação, pois mesmo devidamente traduzidas, podem não pode capturar a riqueza de desafios que uma determinada cultura representa na cognição. Neste artigo vou me concentrar em duas falhas de testes cognitivos disponíveis para AD, sensibilidade ao nível educacional e idade do indivíduo avaliado.

## INTRODUCTION

Accurate diagnosis of Alzheimer's disease (AD) requires a reliable neuropsychological assessment. The criteria that reliable neuropsychological tests for AD should meet have been published in several scientific reports,^1-3^ yet major barriers are still encountered when such tests are used across cultures and during lifespan.^4^ This is particularly problematic in developing countries where most of the available assessment tools have been adapted from developed countries.^5^ This represents a major limitation as these tests, although properly translated, may not embody the wealth of challenges that a particular culture poses on cognition. This paper centers on two shortcomings of available cognitive tests for AD, namely, their sensitivity to the educational background and to the age of the individual assessed. To this end, suggestions and results from recent reports shall be considered, which seem to provide clues to overcoming such limitations.

## EDUCATION AND NEUROPSYCHOLOGICAL ASSESSMENT IN AD

Despite the amount of attention and effort that this topic has drawn from scientists around the globe, this challenge has proven difficult to overcome. Research involving native and immigrant Latino populations has confirmed the notion that education has an impact on cognitive abilities that is above and beyond brain pathology and that valid tests are needed for assessing individuals across a wide range of cultures and education.^5-8^ This need has become more pressing since continuous failures in clinical trials began to be associated with limitations in the cognitive outcome measures available.^9,10^ Predictions are that the growth of dementia cases will be steeper in developing than in developed countries.^11^ Such a future cannot afford the limitations in neuropsychological testing currently faced. Recent studies have started to shed some light on novel cognitive functions that may be less vulnerable to the background education of the population. In a study carried by Parra et al.,^12^ the authors compared two samples of patients with AD drawn from very different populations and diagnosed with different variants of the disease. One sample was from Colombia, South America, and suffered from a genetic variant of AD due to the single mutation E280A in the presenilin-1 gene.^13^ These individuals develop early-onset familiar AD (FAD) at the age of 48 on average. The other sample was from Scotland, UK, and suffered from late-onset sporadic AD (SAD). Both samples were in the early stages of dementia but differed significantly in the number of years spent in formal education, with the Colombian sample having fewer years. The authors reported an equivalent level of impairment across the two samples in a novel cognitive function, namely, short-term memory (STM) binding.

STM binding supports the retention, on a temporary basis, of combinations of features that make up complex objects such as colored shapes. To assess this function, the authors developed a change detection task which asks examinees to briefly hold an array of items and then judge whether a second array shows the same or different items. Items may be made up of a single feature (shape) or of two features (colored shape). The lack of effect of education was also observed when the two control groups of the above-mentioned study, which also differed significantly on the variable, were compared. However, the neuropsychological data reported by the authors reveal a rather different picture. Patients with SAD, who had more years of education and were older (the issue of age is addressed next), were slower than those with FAD on the Trail Making Test part A (TMT-A), performed better on the direct copy of the Complex Figure of Rey-Osterrieth, produced more words following letter clues in the Controlled Oral Word Association Test (COWAT) but fewer following a category clue (Animals). The effect of education on these neuropsychological tasks is well known and seems to add to the effects of age and pathology. The two groups did not differ on the delayed recall of the Complex Figure of Rey-Osterrieth while both were dramatically impaired on this test, scoring 3-4 points out of 36. Studies carried out with Latino populations have consistently shown that performance on the Complex Figure of Rey-Osterrieth task is sensitive to culture and education.^6,7^ Whereas the direct copy of the Complex Figure of Rey-Osterrieth may be more sensitive to education, its recall might reflect effects of the disease beyond the effect of education. Performance on the COWAT showed dissociation across the letter and category task. SAD patients showed no impairment producing words following a letter clue. They outperformed both their local norms and FAD patients. This may reflect the effects of factors such as mild stages of dementia, less vulnerability of letter fluency to age^14,15^ and language.^16^ With regard to this last effect, studies carried out with monolingual (English or Spanish) and bilingual (English and Spanish) populations showed that in the letter clue task these groups produce more English words than Spanish words.^16^ However, SAD patients produced significantly fewer words than FAD patients when the category "Animals" was used as the clue. This result is puzzling since age is known to impact category fluency more than letter fluency^14,15^ and the SAD group was significantly older than the FAD group. Hence, disentangling the particular contribution of age and pathology to this effect proves a challenging task.^17^

What could explain the lack of differences between the two groups on the STM binding test? This task, and other STM binding tasks which have also proved sensitive to SAD and FAD,^18-20^ use non-verbal stimuli. The objects presented during the change detection tasks have neither verbal properties nor representations in long-term memory. Performing such tasks seems to rely on basic visual functions for which literacy may not be relevant. This feature coupled with the simple set of instructions needed (i.e., remember the items on the first screen and decide whether the items that follow are the same or different), makes the task less challenging for people with low education. Moreover, contrary to the neuropsychological tasks discussed above, for which prior experience and cognitive reserves may be critical factors, the STM binding task is not affected by prior knowledge, previous experience or repetition effects.^21^ STM binding seems to be the only integrative memory function that is not disproportionally affected by age.^22,23^ The age-related associative memory deficits hypothesis^24^ states that age widely impacts the ability to retain and learn associations between different pieces of information. This has been confirmed for a wide range of stimuli (e.g. face-name, object-location, word pairs, color-object, etc.). Specifically, the hypothesis proposes that older adults' ability to hold in memory the association between items declines to a much greater extent than their ability to hold the individual items that make up complex experiences. Recent studies on STM binding have consistently demonstrated that processing multiple features bound within object representations in STM is not more sensitive to age than processing the individual features. Hence, contrary to associative memory, memory binding is not disproportionally affected by age. This has proven a feature of the STM binding task that is useful for assessing AD since decline in the function cannot be accounted for by age. This feature is not shared by associative memory tasks.

The neuropsychological data reported by Parra et al.^12^ showed that both SAD and FAD patients were impaired on the TMT-A but that SAD patients were slower than FAD patients. This discrepancy may be accounted for by age, as speed of processing is known to be a marker for cognitive aging.^25^ As discussed above, age extensively impacts cognition and greater interest in cognitive functions that are insensitive to the effects of age has emerged only recently. In the next section, the links between age and cognitive testing in AD shall be addressed while also drawing on the study by Parra et al.^12^'

## AGE AND NEUROPSYCHOLOGICAL ASSESSMENT IN AD

Bondi et al.^26^ showed that the profile of neuropsychological deficits associated with AD in the very-old lacks the disproportionate saliency of episodic memory and executive function deficits typical of the young-old. As people grow older, the boundary between healthy and abnormal aging becomes thinner and this has long delayed the detection of age-related diseases such as dementia.^27^ Most of the available tests of episodic memory functions were designed based on the view that the hippocampus is a structure targeted by AD in its early stages. Hippocampal mediated memory functions decline early in AD but also decline in healthy aging.^28^ For example, of the tasks published by Parra et al.,^12^ the delayed recall of the Complex Figure of Rey-Osterrieth places the greatest demands on hippocampal long-term memory functions.^29^ The pronounced impact of AD on this region may exceed that exerted by age, rendering both groups equally impaired. Unfortunately, this is not always the case, and even with reliable norms, some tasks may not entirely discriminate the proportion of variance of hippocampal functions that is due to age.^30^ As the results of the neuropsychological assessment presented by Parra et al.^12^ suggest, for some cognitive functions the effect of age may be additive only, whereas for others it may interact with that of other factors such as education, leading to an even more complicated cognitive assessment scenario.

Recently, a novel model for assessing and interpreting the effects of AD on cognitive functions has been suggested. Didic et al.,^31^ has proposed a model in which AD first undergoes a sub-hippocampal phase which seems to correspond to Braak's earliest stages (I-III). At this stage, context-rich hippocampal memory functions remain normal but context-free extrahippocampal memory functions start to show impairments. STM binding seems to match the description of tasks of subhippocampal functions as described by Didic et al.^31^ In fact, two recent studies, one in a patient with brain damage^29^ and another involving an fMRI study,^18^ confirmed that STM binding could be performed without an intact hippocampus. This function seems to rely on extrahippocampal regions (e.g. perirhinal and entorhinal cortex) located along the ventral visual stream.^32^ Interestingly, contrary to the hippocampus, which progressively shrinks as people age,^33^ the perirhinal and entorhinal cortex retain their anatomical integrity until very late in life.^34^

## CONCLUSIONS

The effects of culture, education and age represent barriers currently faced in clinical contexts where individuals with dementia or at risk for dementia are routinely assessed. These effects go beyond cultural beliefs regarding aging and dementia^35^ and are limiting the accurate and early detection of AD. Taken together, the research on which this paper has focused,^12^ along with recent suggestions about neuropsychological tasks that are useful for unveiling the preclinical stage of AD,^31^ suggest a shift in the conception of cognitive assessment of Alzheimer's disease is timely (see also^36^). Culturally unbiased cognitive tests are necessary to accumulate comparable data across nations and provide worldwide coverage for pharmaceutical trials. Such tasks should be able to separate the effects of pathology from the effects of other factors such as culture and education. A considerable amount of work has been devoted to addressing this aim over the last few decades.^5^ The results from recent studies suggest that this has been worthwhile in that a promising new generation of cognitive markers for AD is fast approaching.^37^
